# PCNA-binding proteins in the archaea: novel functionality beyond the conserved core

**DOI:** 10.1007/s00294-016-0577-3

**Published:** 2016-02-17

**Authors:** Stuart A. MacNeill

**Affiliations:** Biomedical Sciences Research Complex, School of Biology, University of St Andrews, North Haugh, St Andrews, Fife, KY16 9ST UK

**Keywords:** Sliding clamp, PCNA, Archaea, DNA replication, DNA repair, Chromosome biology, Interactome

## Abstract

Sliding clamps play an essential role in coordinating protein activity in DNA metabolism in all three domains of life. In eukaryotes and archaea, the sliding clamp is PCNA (proliferating cell nuclear antigen). Across the diversity of the archaea PCNA interacts with a highly conserved set of proteins with key roles in DNA replication and repair, including DNA polymerases B and D, replication factor C, the Fen1 nuclease and RNAseH2, but this core set of factors is likely to represent a fraction of the PCNA interactome only. Here, I review three recently characterised non-core archaeal PCNA-binding proteins NusS, NreA/NreB and TIP, highlighting what is known of their interactions with PCNA and their functions in vivo and in vitro. Gaining a detailed understanding of the non-core PCNA interactome will provide significant insights into key aspects of chromosome biology in divergent archaeal lineages.

**Electronic supplementary material:**

The online version of this article (doi:10.1007/s00294-016-0577-3) contains supplementary material, which is available to authorized users.

## Introduction

**Fig. 1:**
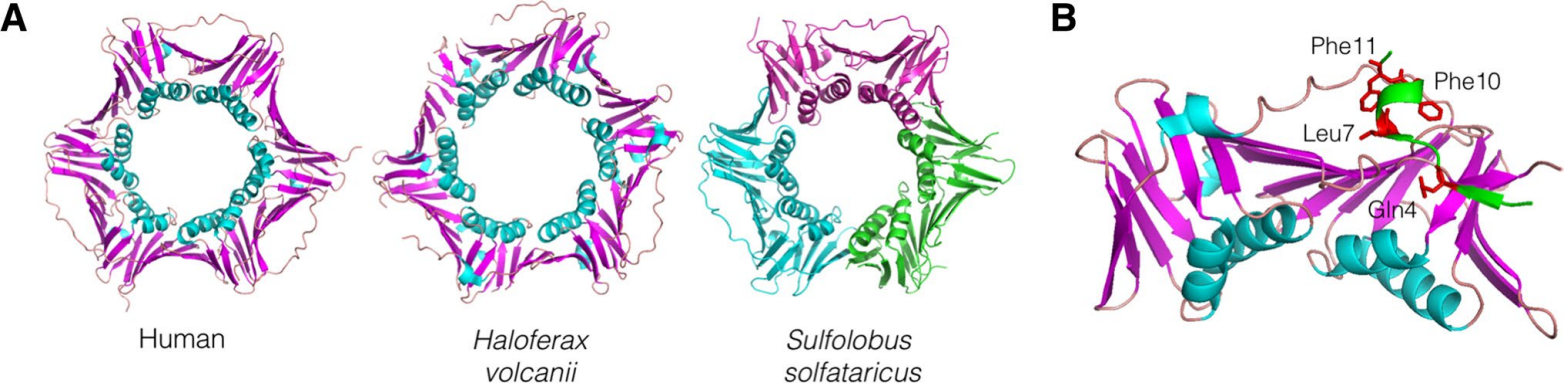
PCNA structure and PIP binding. **a** Structures of homotrimeric PCNA from human cells and the mesophilic halophilic euryarchaeon *Hfx. volcanii*, alongside the heterotrimeric PCNA from the thermophilic crenarchaeon *S. solfataricus*. PDB codes: 4D2G (human), 3HI8 (*Hfx. volcanii*) and 2IX2 (*S. solfataricus*). **b** Details of *A. fulgidus* PCNA showing the binding of a 12-amino acid PIP motif consensus peptide (sequence KTTQSTLDSFFK with conserved residues underlined and labelled on the structure) on the surface. PDB code: 1RXM. See text for details and references

Sliding clamps play crucially important roles in DNA metabolism in all forms of cellular life by encircling double-stranded DNA (dsDNA) and offering a stable platform onto which other factors, typically enzymes with DNA-targeted activities such as DNA polymerases and nucleases, can be assembled in a spatially and temporally coordinated manner. In the archaea, the sliding clamp is PCNA (proliferating cell nuclear antigen), a trimeric protein complex found in either homo- or heterotrimeric forms in different archaeal lineages (Winter and Bunting 2012). Structures of PCNA trimers originating from a variety of archaeal organisms, including homotrimers from the thermophilic euryarchaeal species *Pyrococcus furiosus* (Matsumiya et al. 2001) and *Archaeoglobus fulgidus* (Chapados et al. 2004) and the mesophilic haloarchaeon *Haloferax volcanii* (Morgunova et al. 2009; Winter et al. 2009) as well as the heterotrimer from the thermophilic crenarchaeon *Sulfolobus solfataricus* (Williams et al. 2006), have been solved by crystallography, revealing a striking similarity to one another and also to eukaryotic PCNA (Fig. 1a).

Many of the proteins that interact with PCNA do so via a common conserved sequence motif known as a PIP (PCNA-interacting protein) motif that was first identified in the mammalian DNA replication inhibitor p21^Cip1^ (Warbrick et al. 1995, 1997). The PIP motif is typically defined by the sequence QXXhXXaa, where ‘h’ represents amino acids with moderately hydrophobic side chains (leucine, isoleucine, methionine) and ‘a’ represents amino acids with aromatic hydrophobic side chains (phenylalanine, tyrosine) (Warbrick 1998). PIP motifs have been identified in a number of key highly conserved DNA replication/repair factors including DNA polymerases B and D, replication factor C, the Fen1 nuclease and RNAseH2. Structural studies of co-crystallised archaeal PCNA–PIP peptide complexes have revealed details of the PCNA–PIP interaction in atomic detail (Matsumiya et al. 2002; Chapados et al. 2004). The residues immediately following the well-conserved glutamine (Q) adopt a 3_10_ helix structure than inserts into a hydrophobic pocket formed at the surface of PCNA (Fig. 1b).

The DNA replication/repair factors mentioned above that bind PCNA are central to archaeal DNA replication and repair processes, are highly conserved across different archaeal lineages and can be considered to form the conserved core of the archaeal PCNA interactome. It is becoming increasingly clear, however, that beyond this core group lies a much larger set of PCNA interactors, including proteins of unknown biochemical function, proteins with no homologues outwith the archaea and proteins with very restricted distribution within the archaea. In this review, I summarise recent analysis of three such factors: a dimeric nuclease displaying in vitro activity against a range of DNA substrates (NucS), a representative of a conserved family of archaeal proteins of no known biochemical activity that has been implicated in DNA damage repair (NreA) and a small protein present in a single lineage only that has been proposed as a PCNA inhibitor (TIP).

## NucS: a RecB family nuclease

**Fig. 2:**
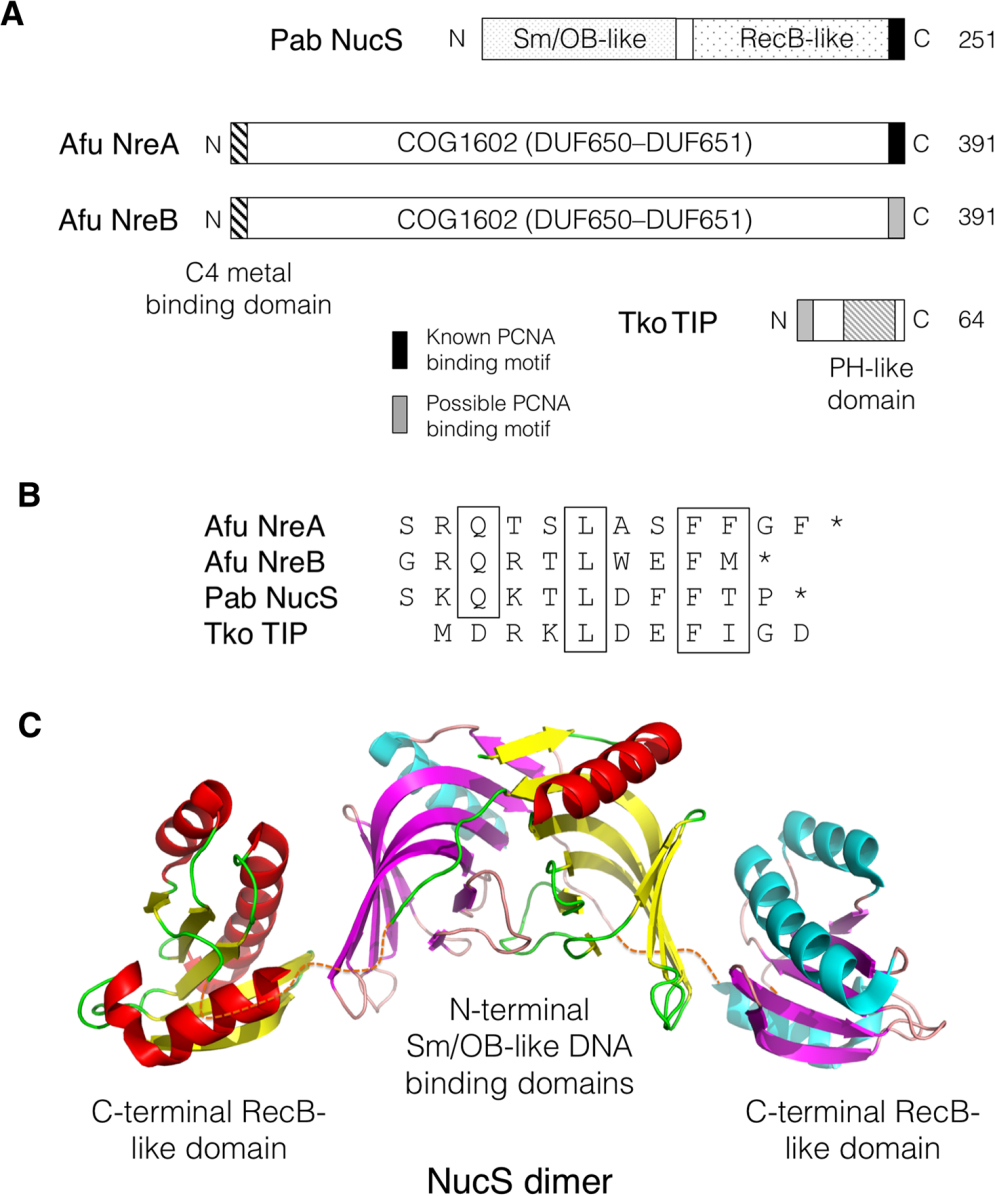
**a** Schematic representation of the *P. abyssi* NucS, *A. fulgidus* NreA and NreB and *T. kodakarensis* TIP proteins highlighting conserved domains. **b** Known (NucS, NreA) or possible (NreB, TIP) PIP motifs in the four proteins with conserved residues boxed (see Supplementary information, Figure S1, for full TIP alignment). **c** Three-dimensional structure of residues 1–233 of the *P. abyssi* NucS protein dimer (PDB code 2VLD). Secondary structure is coloured in *red*/*yellow*/*green* (monomer 1) and *cyan*/*magenta*/*pink* (monomer 2). *Broken lines* indicate the interdomain linker region (residues 115–125) not seen in crystal structure. The C-terminal region (residues 234–251) that includes the PIP motif is also absent from the structure. See text for details and references

The NucS protein was first identified using a combination of bioinformatic searching and peptide array screening for novel PCNA interactors (Meslet-Cladiere et al. 2007). NucS binds with high affinity to PCNA via a C-terminal PIP motif (Fig. 2), shares sequence similarity with the bacterial RecB nuclease family and displays structure-specific nuclease activity on a variety of artificial substrates in vitro (Creze et al. 2012; Ren et al. 2009; Rezgui et al. 2014).

The structure of the 251 amino acid *Pyrococcus abyssi* NucS protein (in isolation, rather than complexed to PCNA) has been solved by crystallography, revealing a dumbbell-like two-domain structure (Fig. 2c) (Ren et al. 2009). The PIP motif is not seen in the structure. The N- and C-terminal domains are separated from each other by a significant distance (almost 30 Å) and are joined by an unstructured linker region. The nuclease active site is located in the C-terminal domain, which is similar to (but much smaller than) the bacterial RecB nuclease domain. The N-terminal domain bares some similarity to the RNA-binding fold of the eukaryotic Sm proteins and the OB fold of ssDNA-binding proteins, suggesting a role in ssDNA binding that was confirmed by mutagenesis studies (Ren et al. 2009). In solution, NucS is a homodimer with the N-terminal domain of one subunit interacting with the C-terminal domain of the other (Fig. 2c). Intriguingly, SAXS (small angle X-ray scattering) analysis of the PCNA–NucS complex in solution indicates that the homotrimeric *P. abyssi* PCNA binds a single NucS dimer only, suggesting that binding somehow alters the symmetrical structure of PCNA to disfavour binding of additional dimers (Creze et al. 2012).

Whilst the in vitro biochemical activity of the NucS protein has been well defined, it is still not known what role the protein plays in vivo, although it has been reported that NucS is non-essential for growth and division in *Hfx. volcanii* (unpublished results cited in Creze et al. 2012). Two lines of evidence point to potential roles for NucS in chromosomal DNA replication and repair (Ren et al. 2009). Pull-down experiments using recombinant NucS and extracts from exponentially growing *P. abyssi* cells identified a small set co-purifying proteins that included—in addition to PCNA—the small and large subunits of the clamp loader complex RF-C, the helicase/nuclease Hef, two subunits of DNA topoisomerase VI and a putative adenine-specific DNA methyltransferase. Further clues to the function of NucS come from the location of the *nucS* gene on the chromosome. In *P. abyssi*, *nucS* is located close to the chromosomal replication origin, whilst in *T. kodakarensis* the *nucS* ORF overlaps that encoding the RadA recombinase (Ren et al. 2009). Taken together, these results hint at an important role for NucS in genome stability without identifying what that role might be. Further work on NucS will be required to address this.

## The Nre proteins: NreA and NreB

The ~400 amino acid Nre proteins (Giroux and MacNeill 2016) are unique to archaea, with no bacterial, eukaryotic or viral Nre homologues apparent in current databases. Almost all sequenced archaeal genomes encode at least one Nre protein (designated NreA) with a C-terminal PIP motif (Fig. 2) but some species also encode a second protein (NreB) with a less clear-cut PIP. Evidence for a direct NreA–PCNA interact comes from a yeast two-hybrid screen that identified the C-terminal domain of *A. fulgidus* NreA as a PCNA interactor (Motz et al. 2002). In addition to possessing a C-terminal PIP motif, many Nre proteins possess an N-terminal Cys4 metal-binding domain that is likely either to comprise a zinc finger or an [Fe–S] cluster (see Fig. 2a). The function of this domain is not known but it is not universally conserved and thus cannot be absolutely essential for Nre protein function (Giroux and MacNeill 2016).

In a number of archaeal genomes, the gene encoding the NreB protein is found adjacent to (and sometimes overlapping with) a gene encoding a protein with significant similarity to bacterial SplB enzymes, suggesting a close functional link (Giroux and MacNeill 2016). SplB (spore photoproduct lyase or SP lyase) is responsible for the repair of a unique type of DNA damage (the intrastrand thymine dimer 5-thyminyl-5,6-dihydrothymine) found exclusively in UV-irradiated bacterial spores. To date, however, there is no evidence for the SP formation in archaea, thus the substrate of the archaeal SplB-like enzymes remains unknown.

To investigate the cellular function of NreA, we used the genetically tractable halophilic euryarchaeon *Hfx. volcanii* as a model system (Giroux and MacNeill 2016). *Hfx. volcanii* encodes a single NreA protein with a C-terminal PIP motif but in common with NreA proteins from other haloarchaea, lacks an N-terminal C4 metal-binding domain. Deletion of the *nraA* gene shows that the NreA protein is not essential for *Hfx. volcanii* cell viability: *∆nreA* cells are indistinguishable from wild type under normal growth conditions and are not supersensitive to the effects of UV irradiation or the alkylating agent methyl methanesulphonate (MMS). They are, however, supersensitive to DNA damage induced by mitomycin C (MMC). MMC reacts with guanine residues to induce three types of DNA lesion: MMC-dG-monoadducts, intrastrand dG-MMC-dG biadducts and interstrand dG-MMC-dG crosslinks. That *∆nreA* cells are sensitive this type of damage suggests a role for the NreA protein in repair of one or more of these lesions.

To analyse this further, we used pulsed-field gel electrophoresis (PFGE) to monitor the structure of *Hfx. volcanii* chromosomes following transient MMC treatment (Giroux and MacNeill 2016). Restriction endonuclease-digested *Hfx. volcanii* chromosomes can be readily resolved by PFGE. Following MMC treatment, the discrete banding is lost and instead, a smear of lower molecular weight DNA is seen. In wild-type cells this is relatively short-lived, with repair of the chromosomes being close to complete 8 h post-exposure. This is not the case in *∆nreA* cells, however, where little or no repair is seen up to 10 h post-exposure.

These results point to a clear role for NreA in repair of MMC damage but do not address the question of whether interaction with PCNA is necessary for this. To address this issue, we used a reverse genetic strategy to create strains in which the NreA protein was expressed in a truncated form lacking the C-terminal PIP motif and tested these for MMC sensitivity and ability to repair chromosomal damage. In both cases, loss of the PIP motif phenocopied complete loss of NreA function, indicating that PCNA binding is essential for NreA’s role in DNA repair (Giroux and MacNeill 2016).

NreA is not the first DNA repair factor to be analysed genetically in *Hfx. volcanii* with previous studies highlighting the roles of the Mre11 and Rad50 proteins (homologues of eukaryotic MRN complex components), the Holliday junction resolvase Hjc, the helicase–nuclease Hef (mentioned above) and UvrA, UvrB, UvrC and UvrD (homologues of bacterial proteins involved in nucleotide excision repair, NER) (Lestini et al. 2010, 2013; Delmas et al. 2009, 2013). We tested whether strains carrying deletions of each of these genes were MMC sensitive and whether deletion of *nreA* in these genetic backgrounds exacerbated this. In the case of *∆mre11 ∆rad50*, *∆hef*, *∆hjc*, *∆uvrA*, *∆uvrB* and *∆uvrC* strains, all six strains displayed increased MMC sensitivity, indicating that each plays a role in repair of MMC-induced DNA damage. Subsequent deletion of *nreA* in *∆mre11 ∆rad50*, *∆hef* and *∆hjc* backgrounds led to an increase in sensitivity, implying that NreA acts in different repair pathways to these factors. However, enhanced sensitivity was not seen when *nreA* was deleted in *∆uvrA*, *∆uvrB* and *∆uvrC* backgrounds, showing that NreA acts together with the UvrABC system to repair MMC-induced DNA damage in *Hfx. volcanii* (Giroux and MacNeill 2016). Similar results were seen when the NreA PIP motif alone was deleted, underlining the key role of PCNA binding in this repair.

Taken together, these results point to Nre proteins potentially playing an important role in DNA damage repair in archaea. What remains to be done? Whilst NreA is clearly important for repair of MMC-induced DNA damage, it is entirely unclear how the protein performs this function and how it interacts with UvrABC. As noted above, every member of the conserved core group of archaeal PCNA-binding proteins is either an enzyme that acts catalytically on DNA or RNA (DNA polymerases B and D, Fen1, RNAseH2) or (in the case of the case of the large subunit of RFC) is part of a complex that binds directly to DNA to perform its catalytic function (PCNA loading). On balance of probability, it seems more likely that the Nre proteins will fall into the first group, i.e. that NreA will act directly on DNA, catalysing strand cleavage or some sort of covalent DNA modification, but analysis of the sequence of the Nre proteins fails to yield any clues as to what the precise function might be. Biochemical analysis of purified Nre proteins is clearly needed to address this. We attempted to take this route with NreA and NreB proteins from a diverse selection of archaeal species but did not succeed (Giroux and MacNeill 2016). In addition, what is the connection between the Nre proteins and the SplB-like proteins encoded by adjacent ORFs in diverse species? Is this an evolutionary relict or an indication of a functional interaction that persists to this day? Indeed, what is the function of the archaeal SplB-like proteins? As far as we are aware, there is no evidence of spore photoproduct formation in archaea, so can we assume that the SplB-like proteins have evolved a different function in DNA repair? It is tempting to speculate, based on the fact that spore photoproduct is an intrastrand crosslink, that this evolved function might be in the repair of a different, more diverse set of intra- or interstrand DNA crosslink structures. Further analysis is clearly required to resolve these issues.

## The TIP protein in the Thermococcales

A third non-core component of the archaeal PCNA interactome is the TIP protein, first identified on the basis of its ability to co-purify with PCNA from extracts prepared from *T. kodakarensis* cells (Li et al. 2014). This organism encodes two distinct homotrimeric PCNA clamps (Kuba et al. 2012; Ladner et al. 2011; Pan et al. 2013)—one essential and one non-essential—and TIP binds to both, albeit with differing affinities. TIP is a small protein (only 64 amino acids) that contains a putative partial PH (pleckstrin homology)-like domain in its C-terminal half (Fig. 2a). Unlike the widely conserved Nre proteins and the reasonably well-conserved NucS, TIP homologues are found only in the Thermococcales, including various *Thermococcus*, *Pyrococcus* and *Palaeococcus* species (see Supplementary information, Figure S1). Genetic analysis of TIP function in *T. kodakarensis* has shown the protein to be non-essential for growth and division and cells lacking TIP to be no more sensitive than wild type to DNA damage induced by UV, MMS or MMC treatment (Li et al. 2014).

Whilst the in vivo function of TIP is unclear, in vitro TIP inhibits PCNA stimulation of PolB activity by inhibiting PCNA–PolB interactions and PCNA stimulation of Fen1, most likely by inhibiting PCNA–Fen1 interactions. How does TIP interact with PCNA? Two regions on the TIP protein have been implicated in PCNA binding by H/D exchange mass spectrometry (HDX-MS), corresponding to amino acids 2–8 and 35–38. TIP was originally reported not to contain a PIP motif and subsequent analysis of the protein was performed in that context (Li et al. 2014). I re-examined this conclusion by performing a multiple sequence alignment of TIP proteins from 26 Thermococcales in current databases (see Supplementary information, Figure S1). Strikingly, the resulting alignment appears to identify a PIP motif at the extreme N-terminus of the TIP proteins, separated from the conserved bulk of the protein (a putative PH-like domain) by a short poorly conserved and potentially unstructured linker (Figure S1). The location of this putative PIP (shown in Fig. 2b) corresponds to the location of the first of the PCNA-binding regions identified by HDX-MS. Whilst we have not undertaken any biochemical analysis of the TIP protein, I would like to propose TIP as a novel PIP motif-containing factor in the archaea and hope that future investigations will test this proposal. The presence of a PIP motif in TIP would provide a simple explanation for the in vitro inhibition of PCNA–PolB and PCNA–Fen1 interactions.

## Concluding remarks

Whilst further analysis is clearly needed to pinpoint the precise molecular functions of the PCNA-interacting proteins discussed in this review, the powerful combination of genetics and biochemistry that can be applied in this arena has already begun to offer fascinating glimpses of the structural and functional diversity to be found beyond the conserved core of the archaeal PCNA interactome.

## Electronic supplementary material

Below is the link to the electronic supplementary material.

Supplementary material 1 (PDF 422 kb)
