# Supplementary material for: PCNA-binding proteins in the archaea: novel functionality beyond the conserved core
Source: Curr Genet. 2016 Feb 17;62:527–32. doi: 10.1007/s00294-016-0577-3 (PMC4929162; doi:10.1007/s00294-016-0577-3)
Supplement: Supplementary file 1 — Supplementary material 1 (PDF 422 kb) [file 294_2016_577_MOESM1_ESM.pdf]

PCNA binding proteins in the archaea: novel functionality beyond the conserved core.

Stuart A. MacNeill

Supplementary information

Figure S1

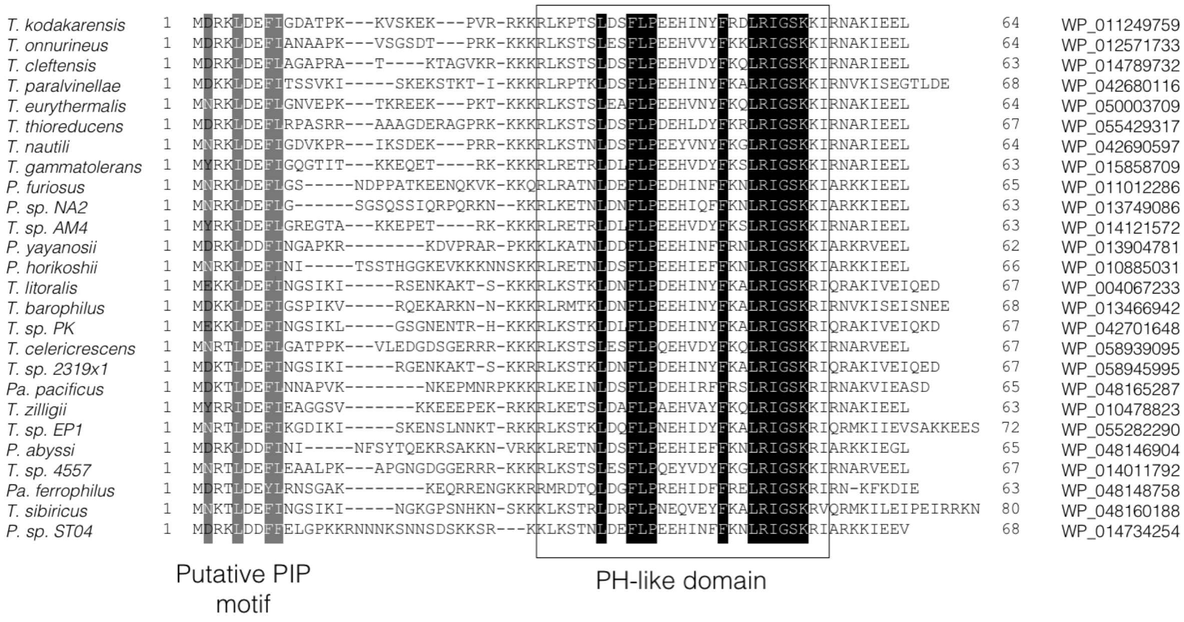

Multiple sequence alignment of TIP proteins from twenty-six species of Thermococcales showing the proposed N-terminal PIP motif (see also Figure 2) and the predicted partial PH-like domain region. Identical residues in the PH-like domain are highlighted in black and conserved residues in the proposed PIP motif residues in grey. Accession numbers are given to the right. Proteins were identified by BLAST searching (Altschul *et al.*, *J. Mol. Biol.* **215**:403-410, 1990) the non-redundant protein sequence database (nr) using the *T. kodakarensis* TIP protein sequence as the query and aligned using COBALT (Papadopoulos and Agarwala, *Bioinformatics* **23**:1073-1079, 2007).
